# Dr. Chapman

**Published:** 1855-03

**Authors:** 


The late Dr. CHAPMAN, of Philadelphia, mourned of many who will laugh at his wit no more, has left behind him a memory that will be transmitted through successive generations. His wit was equal to his skill. It was hard to say which did his patients the most good, and he always gave his best of both at the same time, they probably helped each other. Just as it happened when one of his patients revolted at a monstrous dose of physic, and said:

"Why, Doctor, you don't mean such a dose as this for gentlemen?" "Oh, no," said the Doctor, "it's for *working* men!"

And a good laugh is often as good as a medicine. With him the pleasantry was as certain as the opportunity. Even *in extremis* it would come out of him. He was walking in the streets, and a *baker's* cart, driven furiously, was about to run him down. The baker reined up suddenly, and just in time to spare the Doctor, who instantly took off his hat, and bowing politely, exclaimed, "You are the best *bred* man in town."

At the great gathering in Philadelphia of the Medical Society of the United States, our literary and distinguished Dr. Francis and Dr. Chapman met, as they had done a thousand times before, having been friends for half a century. At a large dinner party a pompous little Dr. Mann, presuming that these gentlemen were strangers, said to Dr. Francis, "Let me introduce you to Dr. Chapman, the head of our profession in Philadelphia." It was too much for Dr. Chapman, who retorted, "Dr. Francis, let me introduce you to Dr. Mann, the *tail* of our profession in Philadelphia." Little Mann let the lions alone after that.

Very much against his will the Doctor was made a vestryman in his parish church, and one of his duties was to pass the plate for the contribution at the morning service. He presented it with great politeness and becoming gravity to the gentleman at the head of the pew nearest the chancel, who was not disposed to contribute. The faithful collector, nothing daunted, held the plate before him, and bowed, as if he would urge him to think the matter over and give *something*, a little something, and refused to go on till he had seen his silver on the plate. In this way he proceeded down the aisle, victimizing every man till he came to the pew nearest the door, where sat an aged colored woman. To his surprise she laid down a piece of gold. "Dear me!" said the astonished Doctor, "you must be a *Guinea* nigger." They never troubled the Doctor to go around with the plate after that.

But we are telling too many of the good things of this good physician. A volume might be made, and a racy one it would be, by any one who would take the trouble to gather up the trifles the Doctor let fall in his public and private walks. One more, and we will leave him.

Dr. Chapman was a delegate to the convention of the church, which was to hold its annual session at Pittsburgh. Party spirit ran high, and the members, both clerical and lay, being men of like passions with other men, became more excited and violent in word and tone than was becoming so reverend and grave a body. When things had gone on at this rate for two days, and were nothing bettered, but rather grew worse, one of the most venerable members arose and said, that he thought these scenes were highly indecorous, especially as they were enacted in the presence of God, whose servants we all pro'ess to be. Dr. Chapman for the first time now stood up, and with a peculiar twisting of his words, and the profound attention of the whole convention, remarked: "Mr. President, I think so too. It is too bad. The members ought not to go on so. But I do not feel the force of that last remark. The gentleman says 'we ought not to conduct in this manner in the presence of God;' now, Sir, to my certain knowledge He has not been in this place since we came together."

The rebuke was so just, so pertinent, that priest and people felt it alike, and the business of the convention was conducted with decorum to its close.

The better half of Dr. Chapman's happy hits were made in the social circles of which he was the life and soul, and they can not be retailed without trenching on the confines of good fellowship, which ought to be sacred against intrusion. Perhaps we have erred on the wrong side in relating some of these. But they are good nevertheless.—*Harper's Magazine.*

